# Modeling the Citation Network by Network Cosmology

**DOI:** 10.1371/journal.pone.0120687

**Published:** 2015-03-25

**Authors:** Zheng Xie, Zhenzheng Ouyang, Pengyuan Zhang, Dongyun Yi, Dexing Kong

**Affiliations:** 1 College of Science, National University of Defense Technology, Changsha, Hunan, China; 2 State Key Laboratory of High Performance Computing, National University of Defense Technology, Changsha, Hunan, China; 3 Department of Mathematics, Zhejiang University, Hangzhou, Zhejiang, China; Universitat Rovira i Virgili, SPAIN

## Abstract

Citation between papers can be treated as a causal relationship. In addition, some citation networks have a number of similarities to the causal networks in network cosmology, e.g., the similar in-and out-degree distributions. Hence, it is possible to model the citation network using network cosmology. The casual network models built on homogenous spacetimes have some restrictions when describing some phenomena in citation networks, e.g., the hot papers receive more citations than other simultaneously published papers. We propose an inhomogenous causal network model to model the citation network, the connection mechanism of which well expresses some features of citation. The node growth trend and degree distributions of the generated networks also fit those of some citation networks well.

## Introduction

Citation network of papers is a directed graph, which describes the inter-citations between the papers. The network regards papers as nodes and contains a directed link from paper *i* to paper *j*, if *i* cites *j*. The idea or method of a paper, more or less, is inspired by its references. The references thus can be treated as sources or causes of the idea or method of the paper. Therefore, the link in citation network is one of causal relationships [2]. Causal relationship extensively exists in physical, biological and social networks [3]. For example, the relationship defined by light cone structure induces a directed graph, called causal network, from universe models [4–6]. Nodes of those networks are sprinkled randomly and uniformly onto spacetimes. Two nodes will be linked by a directed edge from the young node to the old one, if one node is in the other one’s light cone.

D. Krioukov et al proposed the concept of network cosmology in 2012 [7], showing that in- and out-degree distributions in the causal networks of de Sitter space are power-laws and Poissonian respectively [8–10]. Some citation networks [11–16] also have such degree distributions. However, some assumptions of the existing models in network cosmology are not satisfied by various citation networks. For example, in the casual network on a patch of de Sitter space [7], the growth velocity of nodes at time *t* is proportional to cosh(*t*), which is too fast for some empirical data. In addition, the existing casual networks are built on homogenous spacetimes. Hence the nodes born at the same time have equal opportunities to be linked. However, as the empirical studies about ‘attractiveness’ or ‘fitness’ of scientific papers show, the hot papers can receive more links or citations than other contemporaneous papers [17, 18].

We propose an inhomogenous casual network model for citation networks. At each time, we generate a circle, whose center is on a fixed axis, and sprinkle some nodes uniformly and randomly onto this circle. The radius of the circle is proportional to the number of nodes on the circle. Each node attaches an intervals for its angular coordinate, called influence region. Generate a directed link from node *i* to node *j*, if *i*’s angular coordinate belongs to the influence region of *j* and the birth time of *i* is later than that of *j*. The influence region gives a casual relationship for nodes and can be assumed to be inhomogenous: the nodes born simultaneously can have different lengths of influence region.

The connection mechanism is shown to effectively describe the main features of the citing behavior of papers, including relativity, latest, inheritance, popularity, and aging. Assume the growth function of nodes to be an exponential or a constant function of time and the length of the influence region to be inversely proportional to the number of existing nodes. Then the increasing trend of new-born nodes, expected out-degree evolutionary trend, and distributions of the network generated by the model are proved to fit those of some citation networks well.

## The inhomogenous casual network model

**Fig 1:**
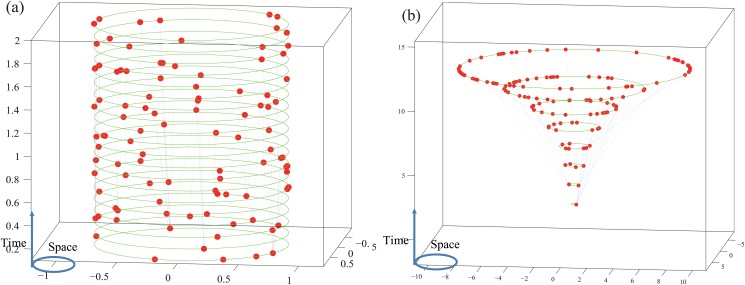
Two networks generated by the CC model. The functions of the CC model are set to be *N*(*t*) = 5, $|D_{i}|=\frac{0.2\beta (\theta_{i})}{t_{i}}$ for the case in Panel(a), and *N*(*t*) = [e^0.1*t*^], $|D_{i}|=\frac{0.15\beta (\theta_{i})}{\left[{\text{e}}^{0.1t_{i}}\right]}$ for the case in Panel(b). *β*(⋅) is given by Equation (2) for both cases.

Consider a (2 + 1)-dimensional spacetime with circumference polar coordinates {*r*, *θ*, *t*}. The nodes of the model are uniformly and randomly sprinkled onto a cluster of circles of the spacetime whose centers are on the time axis(Fig. 1). Hence we name it concentric circles model(CC model). For each time *t* between times *t* = 1 and *t* = *t*_0_,

Step 1. Sprinkle *N*(*t*) nodes uniformly and randomly onto a new circle **S** ^1^ with radius $R(t)=\frac{N(t)}{2\pi \delta }$ centred at point (0, 0, *t*), where *δ* is a positive real number;
Step 2. Give each node *i* an interval *D*_*i*_ for its angular coordinate *θ*_*i*_ to express its influence region;
Step 3. Connect node *i* and node *j* from *i* to *j*, if *θ*_*i*_ ∈ *D*_*j*_ and *t*_*i*_ > *t*_*j*_.

Since the radius coordinates of nodes are not used in what follows, we express nodes by their time and angular coordinates. For node *i* with coordinates (*θ*_*i*_, *t*_*i*_), the arclength of influence region is assumed to be

$$\begin{array}{c} |D_{i}|=\alpha \left(t_{i}\right)\beta \left(\theta_{i}\right), \end{array}$$ (1)

where *α*(*t*_*i*_) is inversely proportional to the number of the existing nodes at time *t*_*i*_, and *β*(*θ*_*i*_) is a piecewise continuous non-negative function of angular coordinate. For example,

$$\begin{array}{c} \beta \left(\theta \right)=\left\{\begin{array}{cc} 4, & \theta \in [0,0.5\pi );\\ 1, & \theta \in [0.5\pi ,2\pi ). \end{array}\right. \end{array}$$ (2)

For citation networks, *α*(⋅) gives a description of the phenomenon that current research is more and more special. *β*(⋅) gives an expression of the inhomogenous popularity of papers published simultaneously.

In this paper, we discuss two type of *N*(*t*): exponential and constant functions. Some journals publish a fixed number of papers at each time. To deal this case, we can assume *N*(*t*) = *m* and

$$\begin{array}{c} |D_{i}|=\frac{\beta (\theta_{i})}{t_{i}}, \end{array}$$ (3)

where *m* is an integer. In some journals, e.g., PNAS, the number of published papers is growing exponentially with time. To model the citation networks from such journals, we assume *N*(*t*) = *m*[e^*lt*^] and

$$\begin{array}{c} |D_{i}|=\frac{\beta (\theta_{i})}{\left[e^{lt_{i}}\right]}, \end{array}$$ (4)

where *m* is an integer, [⋅] is the rounding function, and *l* is a positive real number.

When the influence region is given by Equation (4) and *β*(⋅) is a constant function, the model is a time discrete version of the causal network on a patch of a (1 + 1)-dimensional homogenous spacetime, whose metric in circumference polar coordinates {*t* ∈ [1, *t*_0_], *θ* ∈ ℝ mod 2*π*]} is given by

$$\begin{array}{c} ds^{2}=-dt^{2}+e^{2lt}d\theta^{2}. \end{array}$$ (5)

Metric (5) is a solution of generalized hyperbolic geometric flow [19, 20]. This flow is the resulting equations taking leading terms of the Einstein equations.

**Fig 2:**
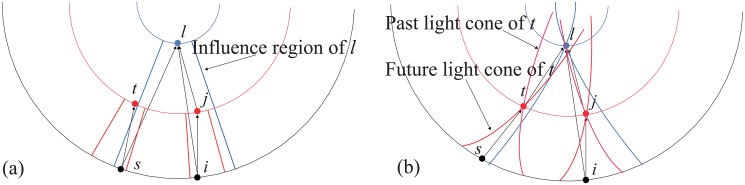
The illustration of the connection mechanisms of the CC model(Panel a) and a causal network on a (1 + 1)-spacetime (Panel b). The influence region of the CC model is the counterpart of the future light cone.

In the causal network of spacetimes, the relationship between nodes is defined by light cone. As Fig. 2 illustrates, the future light cone has a counterpart in the CC model: the influence region, but the past light cone doesn’t have one. In fact, if node *i* belongs to node *j*’s past light cone, then *j* must belong to *i*’s future light cone. Hence the connection relationships given by the past light cones are redundant.

## Modeling the citation behavior

The connection mechanism (Step 3) of the CC model gives geometric expressions to four features of the citing behavior between papers: relativity, latest, inheritance, and popularity.

In order to show details of the sources of the authors’ information, ideas and arguments, it is a basic academic requirement that the papers cite some references which are relevant to themselves. The definition of the influence region expresses the relativity of the nodes: if the angular coordinate of node *i* belongs to *D*_*j*_, we say that *i* is relevant to *j*. Hence the connection mechanism gives a geometric realization that the nodes preferentially connect to the relevant nodes.

Papers cite the latest relevant references. It shows that the authors have a good understanding of recent developments. As Fig. 2(a) shows, the node in the CC model can connect to the latest relevant node.

Paper and the papers it cited usually have some common references. This phenomenon can be called inheritance. In the CC model, the smaller the angular distance Δ(*θ*_*i*_, *θ*_*j*_) = *π* − ∣*π* − ∣*θ*_*i*_ − *θ*_*j*_∣∣ is, the more likely *θ*_*i*_ ∈ *D*_*j*_, and so *i* is relevant to *j*. If the values of Δ(*θ*_*i*_, *θ*_*j*_) and Δ(*θ*_*j*_, *θ*_*l*_) are small, the value of Δ(*θ*_*i*_, *θ*_*l*_) is necessarily small because of the triangle inequality. It means that the probability of *i* ∈ *D*_*l*_ is high. Therefore, the connection behavior of the CC model has the inheritance feature.

Papers prefer to cite the popular or hot papers. Here the node popularity is expressed by the length of node influence region. Since the nodes in the model are distributed uniformly, the nodes with larger influence region have more chances to attract connections. It means that the nodes in the model also prefer connecting to the popular nodes.

The popularity of papers has been fully considered in some typical models for citation networks [21–28]. Those inspiring and effective models focus on fitting the in- and out-degree distributions, clustering coefficients, aging, and assortative property of citation networks. Comparing to those models, as shown in the following sections, the CC model can not only fit the in- and out-degree distributions of some citation networks, but also fit the trends of the annual number of published papers and the trends of the annual average reference lengths of some datasets of papers. In terms of other properties of citation networks, e.g., the abundance of the triangle: paper *i* cites paper *j*, *j* cites *l*, and *i* cites *l* [27], the model of Wu et al [28] can generate a network with a giving a number of triangles that matches the empirical citation networks. The CC model needs to be generalized to have such ability, which is a problem we need to consider in the future.

The relativity of contents is one of the reasons for citation behaviors, which is not fully considered in above models for citation networks. The relativity is called similarity in the Popularity× Similarity optimization model(PSO) [29]. It is an undirected network growth model. In this model, instead of preferring the popular nodes, each new node is connected to a constant number of the existing nodes by optimizing certain trade-off between popularity and similarity. Comparing to the PSO model, the essential difference is that the popularity is inhomogenous in the CC model, but homogenous in the PSO model: the nodes born at the same time has the same popularity.

Inheritance is called copy in the copy model [11]. In this model, a new node attaches to a randomly selected node, as well as all the ancestors of the selected node. It means that if the new node *i* connects to the existing nodes *j* and *l*, there must be a link between *j* and *l*. The CC model does not have this property. In fact, it is a general phenomenon in citation networks that two references cited by one paper may not have a citation between them. In addition, the relativity of the nodes is not considered in the copy model.

## Degree distributions

We calculate the degree distributions for the case whose influence region is defined by Equation (4). The distributions for the other case is the same. The calculation has a little different and is omitted here. For the approximations ‘≈’ in this section, the value of the negligible term is smaller than one tenth of that of the remaining one.

The node with coordinate (*θ*, *t*) belongs to the influence region of the nodes whose coordinates (*ϕ*, *s*) satisfy $\Delta (\theta ,\phi )<\frac{\beta (\phi )}{\left[{\text{e}}^{ls}\right]}$ and *s* < *t*. When $\frac{\beta (\phi )}{\left[{\text{e}}^{ls}\right]}$ is small enough, *β*(*ϕ*) ≈ *β*(*θ*), because that *β*(⋅) is piecewise continuous. Hence the expected out-degree *k* ^+^(*θ*, *t*) of the node with coordinates (*θ*, *t*) is

$$\begin{array}{cc} k^{+}\left(\theta ,t\right) & =\sum_{s=1}^{t-1}\frac{\beta (\theta )}{\left[e^{ls}\right]}R\left(s\right)\delta =\sum_{s=1}^{t-1}\frac{\beta (\theta )}{\left[e^{ls}\right]}\frac{m[e^{ls}]}{2\pi \delta }\delta \approx \frac{\beta (\theta )m}{2\pi }t. \end{array}$$ (6)

**Fig 3:**
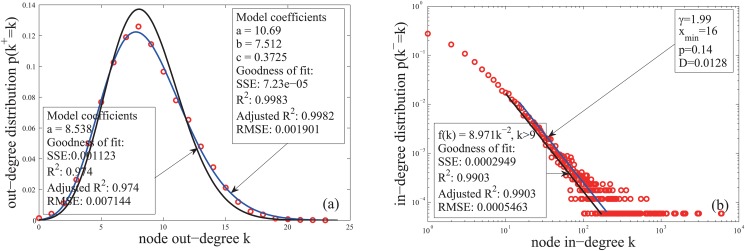
The in- and out-degree distributions of a network generated by the CC model. The functions of the CC model are set as follows: *N*(*t*) = [e^0.1*t*^], $|D_{i}|=\frac{0.15\beta (\theta_{i})}{\left[{\text{e}}^{0.1t_{i}}\right]}$, and *β*(⋅) is given by Equation (2). The fitting functions in Panel (a) are the Poisson distribution $f(k)=a^{k}\frac{{\text{e}}^{-a}}{k!}$ and the mixture Poisson distribution given by Equation (13). The fitting functions in Panel (b) are the power-law functions *f*(*k*) = *ak* ^−2^ and $f(k)=\frac{k^{-\gamma }}{\sum_{n=0}^{\infty }{\left(n+x_{\min}\right)}^{-\gamma }}$.

The approximation holds for *t* > 10. Since the number of nodes increases exponentially with time, the nodes born in times [1, 10] take a small proportion of the total nodes. The expected out-degree of those nodes are small. This makes that the forepart of a fitting curve has a little shifting from the synthetic data of the out-degree distribution(Fig. 3(a)).

The influence region of the node with coordinates (*θ*, *t*) contains the nodes whose coordinates (*ϕ*, *s*) satisfy $\Delta (\theta ,\phi )<\frac{\beta (\theta )}{\left[{\text{e}}^{lt}\right]}$ and *s* > *t*. Hence the expected in-degree *k* ^−^(*θ*, *t*) of the node with coordinates (*θ*, *t*) is

$$\begin{array}{cc} k^{-}\left(\theta ,t\right) & =\sum_{s=t+1}^{t_{0}}\frac{\beta (\theta )}{\left[e^{lt}\right]}R\left(s\right)\delta =\sum_{s=t+1}^{t_{0}}\frac{\beta (\theta )}{\left[e^{lt}\right]}\frac{m[e^{ls}]}{2\pi \delta }\delta \approx \frac{\beta (\theta )m}{2\pi l}\left(e^{l(t_{0}-t)}-1\right)\approx \frac{\beta (\theta )m}{2\pi l}e^{l(t_{0}-t)}. \end{array}$$ (7)

The first approximation holds for e^*lt*^ > 10 and *l* < 1 (approximate e^*l*^ − 1 by the first two terms of its Taylor expansion). The second approximation holds for e^*l*(*t*_0_ − *t*)^ > 10. So the restrictions for time are $t>\frac{1}{l}\log(10)$ and $t_{0}-t>\frac{1}{l}\log(10)$. Since the nodes that don’t satisfy the restrictions are born early or late, the expected in-degree of those nodes are large or small. This makes that the forepart and tail of the fitting curve shift from the synthetic data of the in-degree distribution(Fig. 3(b)).

Since the nodes are distributed according to Poisson point process, the degrees in those networks will not be exactly equal to their excepted values. In order to find the correct in- or out-degree distributions, as Ref. [7] said, we have to average the Poisson distribution,

$$\begin{array}{c} p\left(k^{\pm }\left(\theta ,t\right)=k\right)=\frac{1}{k!}{\left(k^{\pm }\left(\theta ,t\right)\right)}^{k}e^{-k^{\pm }\left(\theta ,t\right)}, \end{array}$$ (8)

which is the probability that node born at time *t* ∈ [1,*t*_0_] has in- or out-degree *k*, with the temporal density *ρ*(*t*). In the CC model,

$$\begin{array}{c} \rho \left(t\right)=\frac{m[e^{lt}]}{\sum_{s=1}^{t_{0}}m\left[e^{ls}\right]}\approx \frac{le^{lt}}{e^{lt_{0}}-1}\propto e^{lt}. \end{array}$$ (9)

of nodes born at time *t*, in which the approximation holds for e^*lt*^ > 10. So the out-degree distribution is the integration

$$\begin{array}{ccc} p(k^{+}=k) & = & \frac{1}{2\pi }\int_{0}^{2\pi }\;\left(\int_{1}^{t_{0}}p\left(k^{+}\left(\theta ,t\right)=k\right)\rho \left(t\right)dt\right)\;d\theta \\ & \propto & \frac{1}{2\pi }\int_{0}^{2\pi }\;\left(\int_{1}^{t_{0}}\frac{{\left(a_{1}t\right)}^{k}e^{-a_{1}t}e^{lt}}{k!}dt\right)\;d\theta \approx \frac{1}{2\pi }\int_{0}^{2\pi }\;\left(a_{1}\int_{a_{1}}^{a_{1}t_{0}}\frac{\tau^{k}e^{-\tau }}{k!}d\tau \right)\;d\theta \\ & = & \frac{1}{2\pi }\int_{0}^{2\pi }a_{1}\;\left(\frac{\Gamma \;\left(k+1,a_{1}\right)-\Gamma \;\left(k+1,a_{1}t_{0}\right)}{k!}\right)\;d\theta \\ & \approx & \frac{1}{2\pi }\int_{0}^{2\pi }\;\left(\frac{a_{1}^{k}e^{-a_{1}}}{k!}\left(1-t_{0}^{k}e^{-a_{1}\left(t_{0}-1\right)}\right)\right)\;d\theta \approx \frac{1}{2\pi }\int_{0}^{2\pi }\left(\frac{a_{1}^{k}e^{-a_{1}}}{k!}\right)\;d\theta , \end{array}$$ (10)

where Γ(⋅, ⋅) is the upper incomplete gamma function, $a_{1}=\frac{\beta (\theta )}{2\pi }$, and *τ* = *a*_1_ *t*. The condition for the first approximation is *a*_1_ > 10*l*, which is satisfied by letting $\frac{\beta (\theta )m}{2\pi }>10l$. We have used lim_*x* → ∞_Γ(*s* + 1, *x*) = *x* ^*s*^e^−*x*^ in the second approximation, which requires a large *a*_1_. The third approximation holds for $t_{0}^{k}{\text{e}}^{-a_{1}(t_{0}-1)}<0.1$, which can be satisfied by setting a large *t*_0_. When *β*(*θ*) is a piecewise constant function, *p*(*k* ^+^ = *k*) is close to a weighted summation of Poisson distributions. This summation is called mixture Poisson distribution.

The in-degree distribution is calculated as follows,

$$\begin{array}{ccc} p(k^{-}=k) & = & \frac{1}{2\pi }\int_{0}^{2\pi }\;\left(\int_{1}^{t_{0}}p\left(k^{-}\left(\theta ,t\right)=k\right)\rho \left(t\right)dt\right)\;d\theta \propto \int_{0}^{2\pi }\;\left(\frac{1}{k!}\int_{a_{2}}^{a_{2}e^{l(t_{0}-1)}}\tau^{k-2}e^{-\tau }d\tau \right)\;d\theta \\ & \approx & \int_{0}^{2\pi }\;\left(\frac{e^{2-k}{\left(k-2\right)}^{k-2}}{k!}\int_{a_{2}}^{a_{2}e^{l(t_{0}-1)}}e^{\;-\frac{{\left(\tau -k+2\right)}^{2}}{2(k-2)}}d\tau \right)\;d\theta \\ & \approx & \frac{1}{k(k-1)}\int_{0}^{2\pi }\;\left(\int_{a_{2}}^{a_{2}e^{l(t_{0}-1)}}\frac{e^{\;-\frac{{\left(\tau -k+2\right)}^{2}}{2(k-2)}}}{\sqrt{2\pi (k-2)}}d\tau \right)\;d\theta , \end{array}$$ (11)

where $a_{2}=\frac{\beta (\theta )m}{2\pi l}$, and *τ* = *a*_2_e^*l*(*t*_0_−*t*)^. Here we have used the Laplace approximation in the third step and the Stirling’s approximation $(k-2)!\approx \sqrt{2\pi (k-2)}{\left(\frac{k-2}{\text{e}}\right)}^{k}$ in the fourth step. The integration in the fourth step is independent of *k* approximately. It can be verified as follows,

$$\begin{array}{cc} \frac{d}{dk}\int_{T_{2}}^{T_{1}}\frac{e^{\;-\frac{{\left(\tau -k+2\right)}^{2}}{2(k-2)}}}{\sqrt{2\pi (k-2)}}d\tau & =\frac{e^{-\frac{{\left(T-k+2\right)}^{2}}{2k-4}}}{2\;\sqrt{2\pi (k-2)}}\left(1+\frac{T}{k-2}\right){|}_{T_{2}}^{T_{1}}\approx 0, \end{array}$$ (12)

where *T*_1_ = *a*_2_e^*l*(*t*_0_−1)^ and *T*_2_ = *a*_2_. The condition for the approximation is a large *a*_2_ or *k*, which is satisfied because of the same reason for the third step of Equation (10). The in-degree distribution is thus a power-law with exponent 2. The numerical experiments (Fig. 3) confirm the results given by Equations (10, 11).

## Fitting the empirical data

In this section, the trends of node growths, the trends of the expected out-degree of nodes, and the degree distributions of some citation data are fitted by above functions respectively. The paraments of the functions are estimated by cftool: a curve fitting toolbox in MATLAB. Four statistical measures: The sum of squares due to error (SSE), Root mean squared error (RMSE), Coefficient of determination (*R* ^2^), and Degree-of-freedom adjusted coefficient of determination (Adjusted *R* ^2^) are used for measuring the goodness of fits.

The in-degree distributions are also fitted by Clauset et al’s method [37]. The fitting function is $f(k)=\frac{k^{-\gamma }}{\sum_{n}^{\infty }=0{\left(n+x_{\min}\right)}^{-\gamma }}$, where *γ* is the scaling exponent and *x*_min_ is the lower bound of the power-law behavior. Here, the parameters *γ* and *x*_min_ are calculated by Clauset et al’s programs (http://tuvalu.santafe.edu/aaronc/powerlaws). The p-value (p) and the maximum distance between the cumulative distribution functions of the data and the fitted function (D) are also calculated by their program to show the goodness of fit tests.

The citation network can only include a subset of the entire papers: if a paper cites, or is cited by, a paper outside the subsets, the network does not contain any information about this. Hence a node’s out-degree is not be exactly equal to the length of its corresponding paper’s references, and its in-degree is also not equal to that in the entire citation network containing the entire papers. We call the in- and out-degrees in the entire network expected in- and out-degrees.

**Fig 4:**
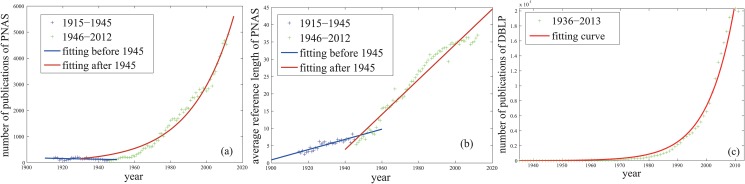
The evolutionary trends of the annual paper number and the annual average reference length of some datasets. The fitting curves for the data between 1946–2012 in Panels(a, b) are *f*(*t*) = 5.397 × 10^−34^e^4.23 × 10^−2^*t*^ (*R* ^2^: 0.974, RMSE: 224.2) and *f*(*t*) = 0.5085*t* − 982.6 (*R* ^2^: 0.958, RMSE: 2.112) respectively. The fitting curve in Panel(c) is *f*(*t*) = 6.038 × 10^−88^e^0.106*t*^ (*R* ^2^: 0.9828, RMSE: 7249).

Consider the dataset for papers from 1915 to 2012 of Proceedings of the National Academy of Sciences(PNAS, http://pnas.org). The first fitness is the exponentially increasing trend of the number of new-born nodes(Equation (9)). It is illustrated in Fig. 4(a) that the number of papers published on PNAS in a given year roughly grows exponentially with time. The annual number of papers in DBLP dataset also roughly shows the exponential increasing trend(Fig. 4(c)).

The second fitness is the linearly increasing trend of the expected out-degree of nodes(Equation (6)). It is illustrated in Fig. 4(b) that the annual average number of references of each PNAS paper grows with time, which is a piecewise linear increasing function of time approximately. The data displays a turn around the year of 1945. So it is cut into two fractions, one is 1915-1945 and the other is 1946-2012 to make a more precise fitting. In our opinion, the main reasons why reference tend to grow slowly or even decline during 1915-1945 is the two world wars(World War I: 1914-1918 and World War II: 1938-1945). During this period, many scientists suffered drift and miserable fates. Many achievements were not published although they did the military a favor. After 1945, the information industry developed so rapidly that all the kinds of science and technology stepped into the golden age. So the relevant prosperity showed in the PNAS dataset in the same period. Obviously, the slope change in 1945 illustrates the development of science after wars. Since the DBLP dataset doesn’t release the information of reference, we won’t analyze the trend of its annual average reference length here. However, the relevant data, the papers from the issues from 1893 to 2003 of Physical Review journals [12], also shows the linearly increasing trend.

The third fitness is the power-law in-degree distributions(Equation (11)). The empirical data (Table 1) includes: the citation networks of papers from e-print arXiv in the period from 1993-01 to 2003-04 in high energy physics phenomenology (Cit-HepPh) and that in high energy physics theory (Cit-HepTh) [13, 14], and the citation networks from DBLP dataset (papers before 2010-05-15, papers before 2013-09-29) collected by Tang et al [15].

**Table 1:** Empirical citation networks.

Network	Nodes	Links
Cit-HepTh	27,770	352,807
Cit-HepPh	34,546	421,578
DBLP 2010-05-15	629,814	632,752
DBLP 2013-09-29	2,084,055	2,244,018

**Fig 5:**
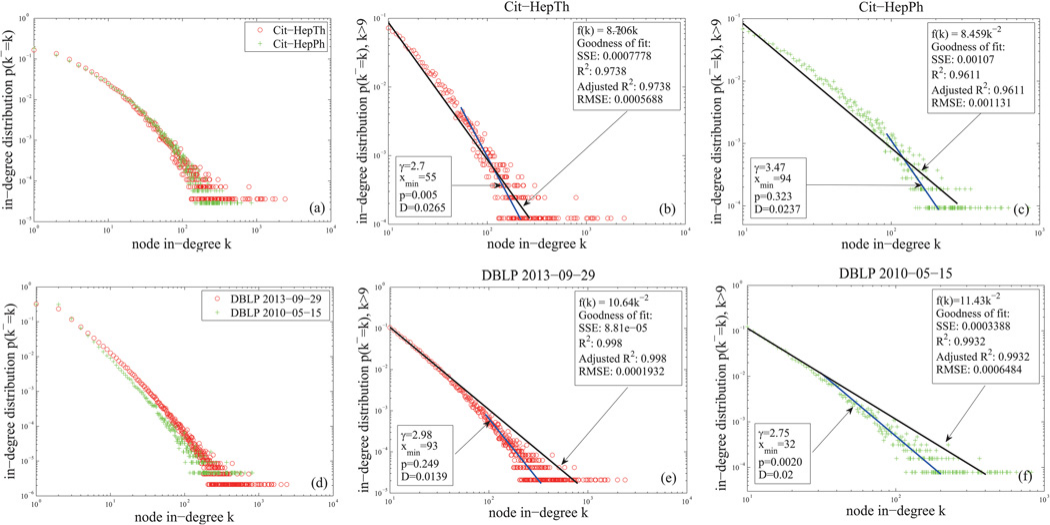
In-degree distributions of the citation networks in Table 1. Panels(b, c, e, f) show the fitting effects of the in-degree distributions of the nodes with in-degree larger than 9 by the power-law functions *f*(*k*) = *ak* ^−2^ and $f(k)=\frac{k^{-\gamma }}{\sum_{n=0}^{\infty }{\left(n+x_{\min}\right)}^{-\gamma }}$.

The statistical measures in Table 2 show that the citation networks from DBLP dataset roughly have the power-law in-degree distributions with power exponent 2, which are similar to the network generated by the CC model(Fig. 3(b)). The in-degree distributions of the nodes with in-degree larger than 9 more accurately fit the power-law distributions with power exponent 2 and the value calculated by the method of Clauset et al [37] (Fig. 5(b, c, e, f)). However, the foreparts of the in-degree distributions of Cit-HepPh and Cit-HepTh do not follow the power-law distributions very well (Fig. 5(a)). The reason for this unfitting phenomenon may be due to the fact that the time scales of these two networks are not large enough (10 years) to meet the CC model demands (the large scale time assumption for the approximations in Equation (11)).

**Table 2:** The goodness for fitting the in-degree distributions of some citation networks by the power-law function *f*(*k*) = *ak* ^−2^.

Network	*a*	SSE	*R* ^2^	Adjusted *R* ^2^	RMSE
Cit-HepTh	0.197	0.02382	0.6354	0.6354	0.003142
Cit-HepPh	0.183	0.02313	0.6031	0.6031	0.005231
DBLP 2010-05-15	0.485	0.007669	0.9707	0.9707	0.003067
DBLP 2013-09-29	0.378	0.01659	0.9028	0.9028	0.002646

As Fig. 5 shows, the curves given by Clauset et al’s method fit the tails of the in-degree distributions better. Hence, we should give the CC model the function for adjusting the power exponent of the in-degree distribution of the generated network. In the next section, we generalize the CC model to model the aging phenomena of the citation behavior. This generalized model has such function.

The fourth fitness is the mixture Poisson distribution for out-degree (Equation (10)). Here we use a simple mixture Poisson distribution to fit the data, which is given by Equation (13),

$$\begin{array}{c} f\left(k\right)=ca^{k}\frac{e^{-a}}{k!}+\left(1-c\right)b^{k}\frac{e^{-b}}{k!}, \end{array}$$ (13)

where *a*, *b* ∈ ℝ, *c* ∈ [0, 1], and *k* ∈ ℤ^+^. The goodness for fit in Table 3 shows that the out-degrees of the citation networks from DBLP dataset approximately follow Equation (13). But the fitting effects for Cit-HepTh and Cit-HepPh are not good. Except for the relatively short time scale, the reason for these unfitting phenomena may be due to the occurrence independence of the Poisson distribution: the events happened in the past have no effect on the probabilities of future occurrences. This kind of independence isn’t fully satisfied in citation networks: papers are more or less effected by the ideas, theories, and methods in the previous papers. The generalized Poisson distribution happens to have the ability to describe the situations where the probability of occurrence of an event is affected by previous occurrences [36].

**Table 3:** The goodness for fitting the out-degree distributions of some citation networks by the mixture Poisson distribution (Equation (13)).*a*, *b*, *c* are parameters of Equation (13).

Network	*a*	*b*	*c*	SSE	*R* ^2^	Adjusted *R* ^2^	RMSE
Cit-HepTh	9.98	1.758	0.5672	0.0144	0.633	0.6306	0.006963
Cit-HepPh	10.48	2.446	0.5734	0.01099	0.709	0.7071	0.006083
DBLP 2010-05-15	0.2404	4.087	0.543	0.003308	0.9843	0.9842	0.00414
DBLP 2013-09-29	0.3283	5.171	0.4777	0.006728	0.9529	0.9525	0.005011

**Fig 6:**
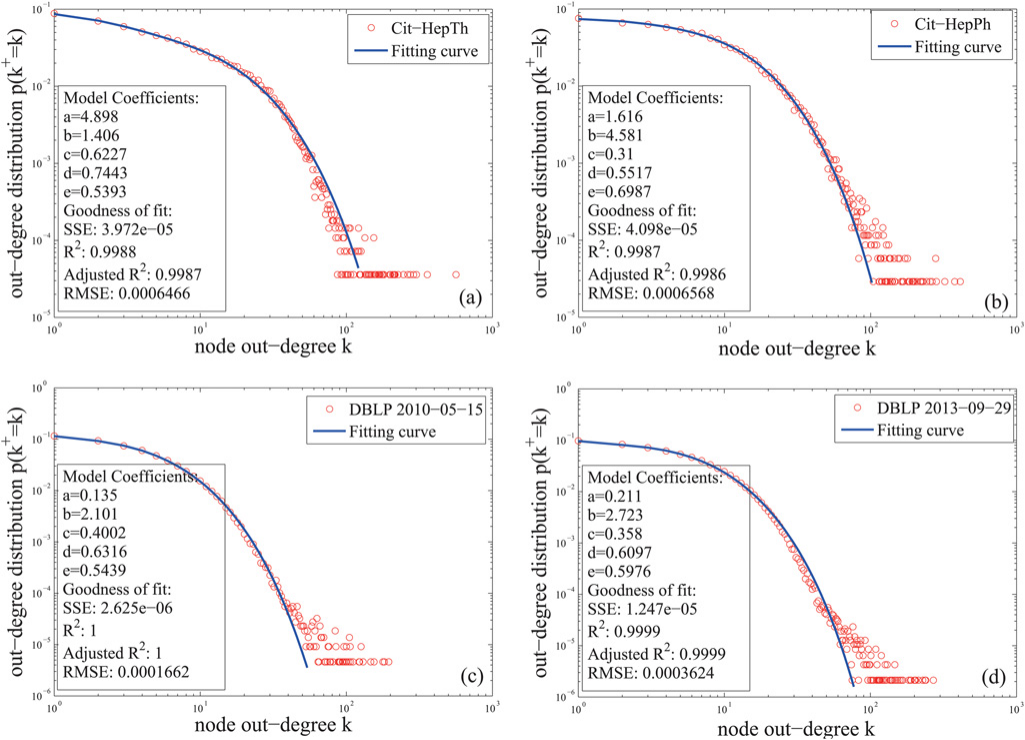
Out-degree distributions of the citation networks in Table 1 and the fitting curves of the distributions. The fitting model is the mixture generalized Poisson distribution (Equation (14)).

We next use the mixture generalized Poisson distribution defined by Equation (14) to fit the out-degree distributions,

$$\begin{array}{c} f\left(k\right)=ca{\left(a+dk\right)}^{k-1}\frac{e^{-a-dk}}{k!}+\left(1-c\right)b{\left(b+ek\right)}^{k-1}\frac{e^{-b-ek}}{k!}, \end{array}$$ (14)

where *a*, *b*, *d*, *e* ∈ ℝ, *c* ∈ [0, 1], and *k* ∈ ℤ^+^. As Fig. 6(a-d) show, the node out-degrees, on the whole, follow the mixture distribution. Meanwhile, the statistical measures in Fig. 6 and in Table 3 show that the fitting effects of Equation (14) are better than Equation (13).

## Modeling the aging phenomena

It has been empirically observed that the probability of a paper to be cited is a decrease function of the paper’s age [30–32]. Some growing network models include the aging of nodes as a feature [33]. In those models, the probability that a paper receives a citation is expressed by a function Γ(*k* ^−^, *t*), which is dependent on the number of citations *k* ^−^ already received and on the publication time *t*. In some models, the two effects are considered to be independent: Γ(*k* ^−^, *t*) = *f*(*k* ^−^)*g*(*t*) with some functional forms of *f*(*k* ^−^) and *g*(*t*) [34, 35].

Under the enlightenment of the fitness expression in the PSO model, we give an influence region with aging effect: the influence region length of node *i* with coordinates (*θ*_*i*_, *t*_*i*_) is given by

$$\begin{array}{c} |D_{i}|=\frac{\beta (\theta_{i})}{\left[e^{l(at_{i}+\left(1-a\right)t_{c})}\right]}, \end{array}$$ (15)

where *l* > 0, *t*_*c*_ is the current time, and *a* ∈ [0, 1) is a parameter tuning the velocity of aging. When *a* > 0, the length of the node influence region is a decreasing function of *t*_*c*_, which models the phenomena that the probability of papers to be cited decreases with the papers’ age.

When the influence region is given by Equation (15), the expected in- and out-degree of the node with coordinate (*θ*, *t*) is

$$\begin{array}{ccc} k^{-}\left(\theta ,t\right) & = & \sum_{s=t+1}^{t_{0}}\frac{\beta (\theta )}{\left[e^{l(at+(1-a)s)}\right]}R\left(s\right)\delta \approx \frac{\beta (\theta )m}{2\pi }\int_{t}^{t_{0}}e^{la(s-t)}ds\approx \frac{\beta (\theta )m}{2\pi la}e^{la(t_{0}-t)}, \end{array}$$ (16)

$$\begin{array}{ccc} k^{+}\left(\theta ,t\right) & = & \sum_{s=1}^{t-1}\frac{\beta (\theta )}{\left[e^{l((1-a)t+as)}\right]}R\left(s\right)\delta \approx \frac{\beta (\theta )m}{2\pi }\int_{0}^{t-1}e^{l(1-a)(s-t)}ds\\ & \approx & \frac{\beta (\theta )m}{2\pi }\frac{e^{-l(1-a)}-e^{-l(1-a)t}}{l(1-a)}. \end{array}$$ (17)

The approximations hold for lager *t* and *t*_0_.

When *t* is lager enough, *k* ^+^(*θ*, *t*) tends to a function which is free of *t*. It has been empirically observed that the annual average number of paper references is a monotone increasing sequence for some journals, e.g., PNAS(Fig. 4(a)). Meanwhile, it is reasonable to think that the number of paper references can’t grow to infinity, and should have an upper bound. Hence, the expected out-degree given by Equation (17) is reasonable, because that a bounded monotonic sequence has a limit.

With the similar calculations as those in Equations (10, 11), we find that the network generated by the model whose influence region is given by Equation (15) has a power-law distribution with exponent $1+\frac{1}{a}$ for in-degree. The out-degree distribution is close to a mixture Poisson distribution.

## Conclusions

We propose a model for citation networks using network cosmology, whose connection mechanism gives a geometric expression of the main features of the citing behaviors: relativity, latest, inheritance, popularity, and aging. The model generalizes the homogenous assumption of some existing models in network cosmology: the nodes born at the same time can have different popularity. This property gives an expression of the phenomenon that hot papers can receive more citations than other concurrent published papers. We show that the node growth trend, expected node out-degree, and degree distributions of the network generated by the model fit those of some citation networks well.

## References

[1] Kenneth DB (2005) Fifty Years of Systems Science: Further Reflections. Syst Res Behav Sci 22: 355–361. 10.1002/sres.711

[2] Groff R (2008) Revitalizing Causality: Realism about Causality in Philosophy and Social Science. Routledge, New York.

[3] Dorogovtsev SN, Mendes JFF (2003) Evolution of Networks: From Biological Nets to the Internet and WWW. Oxford University Press, Oxford.

[4] Ahmed M, Rideout D (2010) Indications of de Sitter spacetime from classical sequential growth dynamics of causal sets, Phys Rev D 81: 083528 10.1103/PhysRevD.81.083528

[5] Bombelli L, Lee J, Meyer D, Sorkin R (1987) Spacetime as a causal set. Phys Rev Lett 59: 521–524. 10.1103/PhysRevLett.59.521 10035795

[6] Rideout D, Sorkin R (1999) Classical sequential growth dynamics for causal sets. Phys Rev D 61: 024002 10.1103/PhysRevD.61.024002

[7] Krioukov D, Kitsak M, Sinkovits RS, Rideout D, Meyer D, Boguñá M (2012) Network Cosmology. Sci Rep 2:793 10.1038/srep00793 23162688 PMC3499772

[8] Papadopoulos F, Psomas C, Krioukov D (2012) Replaying the geometric growth of complex networks and application to the as internet. Perform Eval Rev 40: 104–106. 10.1145/2425248.2425277

[9] Boguñá M, Kitsak M, Krioukov D (2013) Cosmological networks. arXiv:abs/1310.6272.

[10] Krioukov D, Papadopoulos F, Kitsak M, Vahdat A, Boguñá M (2010) Hyperbolic geometry of complex networks. Phys Rev E 82: 36106 10.1103/PhysRevE.82.036106 21230138

[11] Krapivsky PL, Redner S (2005) Network Growth by Copying. Phys Rev E 71: 036118 10.1103/PhysRevE.71.036118 15903504

[12] Redner S (2004) Citation statistics from more than a century of physical review. arXiv:physics/0407137.

[13] Leskovec J, Kleinberg J, Faloutsos C (2007) Graph Evolution: Densification and Shrinking Diameters. ACM TKDD 1(1): 2 10.1145/1217299.1217301

[14] Gehrke J, Ginsparg P, Kleinberg J (2003) Overview of the 2003 KDD Cup. SIGKDD Explorations 5: 149–151. 10.1145/980972.980992

[15] Tang J, Zhang J, Jin RM, Yang Z, Cai KK, Zhang L, et al (2011) Topic level expertise search over heterogeneous networks. Mach Learn J 82: 211–237. 10.1007/s10994-010-5212-9

[16] Börner K, Maru TJ, Goldstone RL (2004) The simultaneous evolution of author and paper networks, Proc Natl Acad Sci USA 101: 5266–5273. 10.1073/pnas.0307625100 14976254 PMC387306

[17] Eom YH, Fortunato S (2011) Characterizing and modeling citation dynamics. PLoS ONE 6(9): e24926 10.1371/journal.pone.0024926 21966387 PMC3178574

[18] Wang D, Song C, Barabási AL (2013) Quantifying long-term scientific impact. Science 342: 127–131. 10.1126/science.1237825 24092745

[19] Kong DX, Liu KF (2007) Wave character of metrics and hyperbolic geometric flow. J Math Phys 48:103508 10.1063/1.2795839

[20] Kong DX, Liu KF, Xu DL (2009) The hyperbolic geometric flow on Riemann surfaces. Commun Part Diff Eq 34: 553–580. 10.1080/03605300902768933

[21] Price DJ de Solla (1965) Networks of scientific papers. Science 149(3683): 510–515. 10.1126/science.149.3683.510 14325149

[22] Price DJ de Solla (1976) A general theory of bibliometric and other cumulative advantage process. J Am Soc Inf Sci 27(5): 292–306. 10.1002/asi.4630270505

[23] Rednes S (1998) How popular is your paper? An empirical study of the citation distribution. Eur Phys J B 4(2):131–134. 10.1007/s100510050359

[24] Karrer B, Newman MEJ (2009) Random acyclic networks. Phys Rev Lett 102(12): 128701 10.1103/PhysRevLett.102.128701 19392330

[25] Karrer B, Newman MEJ (2009) Random graph models for directed acyclic networks. Phys Rev E 80(4): 046110 10.1103/PhysRevE.80.046110 19905393

[26] Milo R, Shen-Orr S, Itzkovitz S, Kashtan N, Chklovskii D, Alon U (2002) Network motifs: Simple building blocks of complex networs. Science 298(5594): 824–827. 10.1126/science.298.5594.824 12399590

[27] Chen P, Xie H, Maslov S, Redner S (2007) Finding scientific gems with Google. J Informetr 1(1):8–15. 10.1016/j.joi.2006.06.001

[28] Wu ZX, Holme P (2009) Modeling scientific-citation patterns and other triangle-rich acyclic networks. Phys Rev E 80(3): 037101 10.1103/PhysRevE.80.037101 19905247

[29] Papadopoulos F, Kitsak M, Serrano MA, Boguñá M, Krioukov D (2012) Popularity versus similarity in growing networks. Nature 489: 537–540. 10.1038/nature11459 22972194

[30] Hajra KB, Sen P (2004) Phase transitions in an aging network. Rhys Rev E 70(5): 056103 10.1103/PhysRevE.70.056103 15600688

[31] Hajra KB, Sen P (2005) Modeling aging characteristics in citation networks. Physica A 368(2): 575–582. 10.1016/j.physa.2005.12.044

[32] Wang M, Yu G, Yu D (2008) Measuring the preferential linear attachment mechanism in citation networks. Physica A 387(18): 4692–4698. 10.1016/j.physa.2008.03.017

[33] Radicchi F, Fortunato S, Vespignani A, Citation Networks (2012) In: Scharnhorst A, Börner K, Besselaar PVD editors. Models of science dynamics. Springer pp. 233–257.

[34] Dorogovtsev SN, Mendes JFF (2000) Evolution of networks with aging of sites. Phys Rev E 62(2): 1842–1845. 10.1103/PhysRevE.62.1842 11088645

[35] Zhu H, Wang X, Zhu JY (2003) Effect of aging on network structure. Phys Rev E 68(5): 056121 10.1103/PhysRevE.68.056121 14682860

[36] Consul PC, Jain GC (1973) A generalization of the Poisson distribution Technometrics 15(4): 791–799.

[37] Clauset A, Shalizi CR, Newman MEJ (2009) Power-law distributions in emprical data. SIAM Rev 51: 661–703. 10.1137/070710111

