# Correction: Modeling the Citation Network by Network Cosmology

**DOI:** 10.1371/journal.pone.0140413

**Published:** 2015-10-30

**Authors:** The PLOS ONE Staff

Figs 1, 2, 3, 4, 5 and 6 are incorrect. The figures should be formatted as EPS files. The publisher apologizes for the error. The authors have provided a corrected version here.

**Fig 1:**
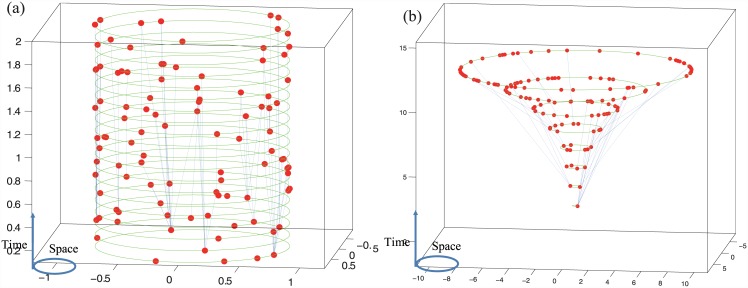
Two networks generated by the CC model. The functions of the CC model are set to be N(t) = 5, ∣Di∣ = 0.2β(θi)ti for the case in Panel(a), and N(t) = [e0.1t], ∣Di∣ = 0.15β(θi)[e0.1ti] for the case in Panel(b). β(⋅) is given by Equation (2) for both cases.

**Fig 2:**
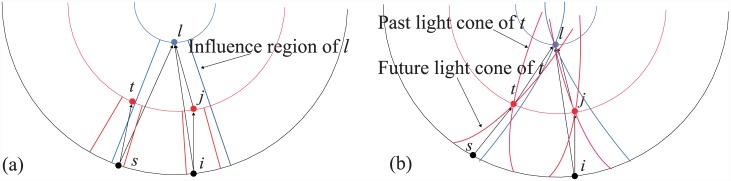
The illustration of the connection mechanisms of the CC model(Panel a) and a causal network on a (1 + 1)-spacetime (Panel b). The influence region of the CC model is the counterpart of the future light cone.

**Fig 3:**
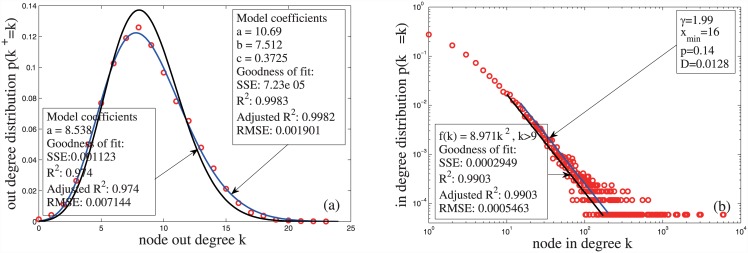
The in- and out-degree distributions of a network generated by the CC model. The functions of the CC model are set as follows: *N*(*t*) = [e^0.1t^], ∣Di∣ = 0.15β(θi)[e0.1ti], and *β*(⋅) is given by Equation (2). The fitting functions in Panel (a) are the Poisson distribution f(k) = ake−ak! and the mixture Poisson distribution given byEquation (13). The fitting functions in Panel (b) are the power-law functions *f*(*k*) = *ak* ^−2^ and f(k) = k−γ∑n = 0∞(n+xmin)−γ.

**Fig 4:**
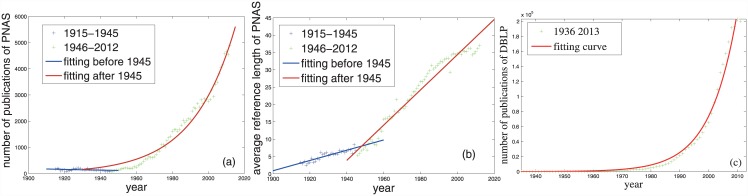
The evolutionary trends of the annual paper number and the annual average reference length of some datasets. The fitting curves for the data between 1946–2012 in Panels(a, b) are *f*(*t*) = 5.397 × 10^−34^e^4.23 × 10−2 t^ (*R* ^2^: 0.974, RMSE: 224.2) and *f*(*t*) = 0.5085*t* − 982.6 (*R* ^2^: 0.958, RMSE: 2.112) respectively. The fitting curve in Panel(c) is *f*(*t*) = 6.038 × 10^−88^e^0.106t^(*R* ^2^: 0.9828, RMSE: 7249).

**Fig 5:**
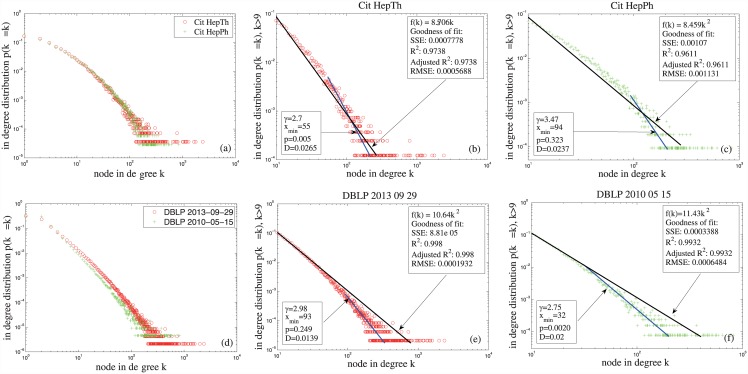
In-degree distributions of the citation networks in Table 1. Panels(b, c, e, f) show the fitting effects of the in-degree distributions of the nodes with in-degree larger than 9 by the power-law functions *f*(*k*) = *ak* ^−2^ and f(k) = k−γ∑n = 0∞(n+xmin)−γ.

**Fig 6:**
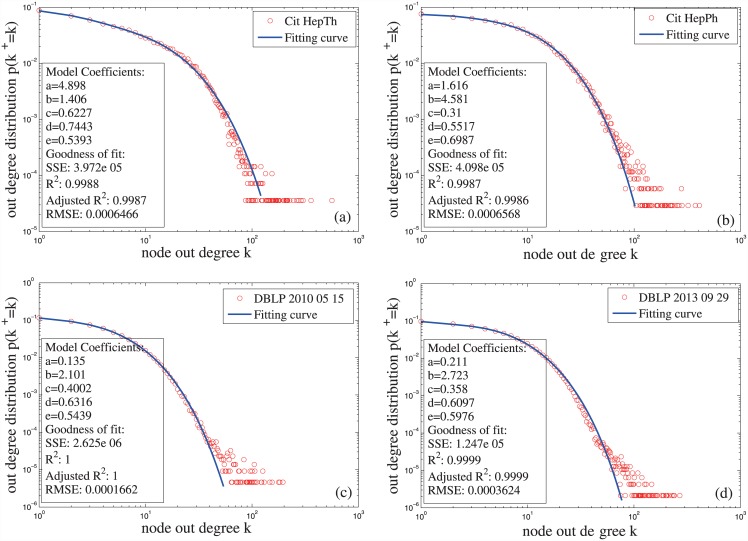
Out-degree distributions of the citation networks in Table 1 and the fitting curves of the distributions. The fitting model is the mixture generalized Poisson distribution (Equation (14).
